# The π-Electron Delocalization in 2-Oxazolines Revisited: Quantification and Comparison with Its Analogue in Esters

**DOI:** 10.3390/ma8085249

**Published:** 2015-08-21

**Authors:** Martin Fimberger, Klaus P. Luef, Claudia Payerl, Roland C. Fischer, Franz Stelzer, Mihály Kállay, Frank Wiesbrock

**Affiliations:** 1 Polymer Competence Center Leoben, Roseggerstrasse 12, 8700 Leoben, Austria; E-Mails: martin.fimberger@pccl.at (M.F.); klauspeter.luef@pccl.at (K.P.L.); 2 Institute for Chemistry and Technology of Materials, Graz University of Technology, NAWI Graz, Stremayrgasse 9, 8010 Graz, Austria; E-Mails: claudia.payerl@tugraz.at (C.P.); franz.stelzer@tugraz.at (F.S.); 3 Institute of Inorganic Chemistry, Graz University of Technology, NAWI Graz, Stremayrgasse 9, 8010 Graz, Austria; E-Mail: roland.fischer@tugraz.at; 4 MTA-BME Lendület Quantum Chemistry Research Group, Department of Physical Chemistry and Materials Science, Budapest University of Technology and Economics, P.O. Box 91, 1521 Budapest, Hungary; E-Mail: kallay@mail.bme.hu

**Keywords:** 2-oxazoline monomers, π-electron delocalization, regioselectivity of the initiation of cationic ring-opening polymerizations, ester-functionalized 2-oxazoline, hydrolysis of 2-oxazoline, methyl 3-(4,5-dihydrooxazol-2-yl)propanoate

## Abstract

The single crystal X-ray analysis of the ester-functionalized 2-oxazoline, methyl 3-(4,5-dihydrooxazol-2-yl)propanoate, revealed π-electron delocalization along the N–C–O segment in the 2-oxazoline pentacycle to significant extent, which is comparable to its counterpart along the O–C–O segment in the ester. Quantum chemical calculations based on the experimental X-ray geometry of the molecule supported the conjecture that the N–C–O segment has a delocalized electronic structure similar to an ester group. The calculated bond orders were 1.97 and 1.10 for the N=C and C–O bonds, and the computed partial charges for the nitrogen and oxygen atoms of −0.43 and −0.44 were almost identical. In the ester group, the bond orders were 1.94 and 1.18 for the C–O bonds, while the partial charges of the oxygen atom are −0.49 and −0.41, which demonstrates the similar electronic structure of the N–C–O and O–C–O segments. In 2-oxazolines, despite the higher electronegativity of the oxygen atom (compared to the nitrogen atom), the charges of the hetero atoms oxygen and nitrogen are equalized due to the delocalization, and it also means that a cationic attack on the nitrogen is possible, enabling regioselectivity during the initiation of the cationic ring-opening polymerization of 2-oxazoline monomers, which is a prerequisite for the synthesis of materials with well-defined structures.

## 1. Introduction

**Scheme 1:**
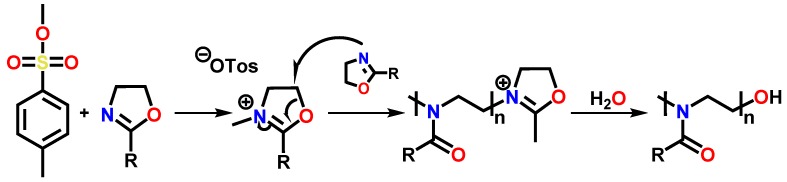
Methyl tosylate-initiated polymerization of 2-oxazolines.

Since the introduction of microwave reactors dedicatedly designed for usage in laboratories, considering the chemists’ and material scientists’ requirements [1,2,3,4], the class of poly(2-oxazoline)s has been reawakened from its hibernation (Scheme 1): discovered in 1966/67 by four research groups almost simultaneously [5,6,7,8], the versatility of this class of polymers, and its derived materials, has been under constant investigation, while the commonly-low polymerization rates have been the bottleneck for a long time in terms of applicability of these materials. With the exclusion of acceleration-limiting factors such as temperatures (boiling points), microwave reactors have helped to successfully accelerate the polymerizations of 2-oxazolines, and, currently, they are under thorough investigation, in particular, for usage in medical and sanitary applications [9,10,11,12,13,14,15] and as cross-linkable materials [16,17,18,19,20,21,22].

In particular for medical and medicinal applications, a precise knowledge of the materials’ structures is of key importance, which makes living or at least pseudo-living polymerizations and their inherent access to polymers with narrow molecular weight distributions favorite synthetic strategies. For the polymerization of 2-oxazolines, it has been shown that the highly reactive methyl tosylate is one of the initiators that can start pseudo-living polymerizations [23,24,25]. Due to its high reactivity, it has been argued whether the initiation by methyl cations occurs regioselectively at the nitrogen atom (Scheme 1) when the polymerization times experience accelerations by a factor of up to 400 [26].

**Scheme 2:**
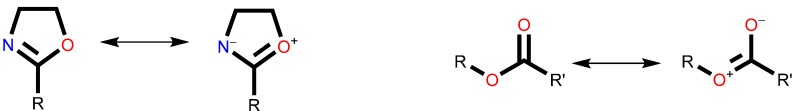
Delocalization of π-electrons in 2-oxazolines (**left**) and esters (**right**).

In a precedent study [27], we could show that, due to π-electron delocalization, the partial negative charge at the oxygen atom of the 2-oxazoline ring is lessened (Scheme 2). The negative charge of the nitrogen atom, on the other hand, is enhanced and, hence, the nitrogen atom is an ideal reaction partner for the methyl tosylate. In order to expand the understanding of this π-electron delocalization in 2-oxazolines, we aimed for a correlation/comparison with its counterparts in esters, where the C–O “single” bond as well has been reported to show an intermediate value between that of a C–O double and single bond. In this study, we therefore present the single crystal X-ray analysis of an ester-functionalized 2-oxazoline and the corresponding ring-opened ester-functionalized amino acid.

## 2. Experimental Section

### 2.1. Materials

All chemicals were used as received. Chloroethylamine hydrochloride, hydrochloric acid solution (0.1 mol/L), methyl chlorobutyrate, triethylamin, and calcium carbonate were purchased from Sigma Aldrich (Vienna, Austria), while ethanol, methanol, dichloromethane, and sodium hydroxide were bought from Carl Roth (Karlsruhe, Germany).

### 2.2. Instrumentation

IR spectra were recorded with 48 scans per sample on a Bruker Alpha FT-IR spectrometer (Bruker Optics Inc., Billerica, MA, USA) equipped with the ALPHA’s Platinum attenuated total reflection (ATR) single reflection diamond ATR module. The spectral range was set from 500 to 4000 cm^−1^. ^1^H NMR spectra were measured in deuterated chloroform or deuterium dioxide on a Bruker 300 MHz spectrometer (Bruker BioSpin Corporation, Billerica, MA, USA) with 32 scans and relaxation delays of 5 s. The solvent residual peaks were used for referencing the spectra to 7.26 ppm and 4.80 ppm, respectively.

### 2.3. Single Crystal X-ray Diffraction Analyses

The crystalline samples were placed in inert oil, mounted on a glass pin and transferred to the cold gas stream of the diffractometer. Crystal data were collected and integrated with a Bruker APEX-II CCD system (Bruker AXS GmbH, Karlsruhe, Germany) with monochromated Mo-K_α_ (λ = 0.71073 Å) radiation at 100(2) K. The structures were solved by direct methods using SHELXS-97 [28] and refined by full matrix least squares calculations on *F*^2^ with SHELXL-97 [29]. The space group assignments and structural solutions were evaluated using PLATON [30]. Non-H-atoms were refined with anisotropic thermal parameters. All protons located on carbon atoms were calculated and allowed to ride on their parent atoms with fixed isotropic contributions; protons on nitrogen atoms were located and refined with isotropic contributions. Extinction corrections were applied for all compounds using SADABS [31]. A summary of the crystal data, experimental details and refinement results is listed in Table 1. Important interatomic distances and angles are given in the figure captions. Thermal parameters and complete tables of interatomic distances and angles have been deposited with the Cambridge Crystallographic Data Centre, 12 Union Road, Cambridge CB2 1 EZ, UK. The data are available on request on quoting CCDS-1418758/1418759 and in the Supporting Information.

**Table 1:** Crystal data, data collection, absorption and structure refinement of methyl 3-(4,5-dihydrooxazol-2-yl)propanoate **EstOx** (ester-functionalized 2-oxazoline) and 4-(2-aminoethoxy)-4-oxobutanoic acid **EstAA** (ester-functionalized amino acid).

Crystal Structure Analysis	Methyl 3-(4,5-dihydrooxazol-2-yl) Propanoate, EstOx	4-(2-Aminoethoxy)-4-oxo-butanoic Acid, EstAA
**Crystal data**		
CCDC No.	1418758	1418759
Crystal form	block	block
Crystal colour	colorless	colorless
Empirical formula	C_7_H_11_NO_3_	C_6_H_11_NO_4_
Formula weight	157.17	161.16
Crystal system	monoclinic	monoclinic
Space group	*P*2_1_	*P*2_1_/c
*a* (Å)	5.547 (2)	10.3132 (8)
*b* (Å)	6.765 (3)	9.0304 (7)
*c* (Å)	9.993 (4)	8.0012 (7)
α (°)	90	90
β (°)	91.583 (13)	96.688 (5)
γ (°)	90	90
*V* (Å^3^)	374.9 (3)	740.10 (10)
ρ_calc_ (g·cm^−3^)	1.268	1.446
Z	2	4
*F*(0 0 0)	152	344
µ (Mo-K_α_) (cm^−1^)	0.102	0.122
**Data Collection**		
Measured reflections	4559	1786
Unique reflections	1278	1786
*R*_int_	0.0659	0.000
**Absorption**		
*T*_min_/*T*_max_	0.9690/0.9808	0.9574/0.9855
**Refinement Results**		
Refined parameters	101	101
*R*_1_^a,b^, *wR*_2_^a;c^	0.0530; 0.1352	0.0630; 0.1432
*a*, *b*	0.0887; 0.0457	0,000; 2.2816
ρ (e·Å^−3^)	0.306; −0.219	0.362; −0.325

^a^ *I* > 2σ(*I*); ^b^ *R*_1_ = Σ(||*Fo*| − |*Fc*||/Σ|*Fo*|; ^c^ *wR*_2_ = {[Σ*w*(*Fo*^2^ − *Fc*^2^)2]/Σ[*w*(*Fo*^2^)^2^]}^0.5^; *w* = 1/[σ^2^(*Fo*^2^) + (a*p*)^2^ + b*p*]; p = (*Fo*^2^ + 2*Fc*^2^)/3; a and b: free variables.

### 2.4. Preparation of Methyl 3-(4,5-Dihydrooxazol-2-yl)propanoate **EstOx**

Ten millilitres (0.13 mol) of methyl 4-chloro-4-oxobutanoate and 15.41 g (0.13 mol) of chloroethylamine hydrochloride were dissolved in 130 mL of dichloromethane under inert conditions and cooled to 0 °C. Forty-one millilitres of triethylamine, dissolved in 20 mL of dichloromethane, were added dropwise within 1 h, and the reaction mixture was stirred overnight. The organic phase was extracted twice with deionized water and once with brine, prior to drying with sodium sulphate. Subsequently, the solvent was removed under reduced pressure. 17.40 g of the dry intermediate product were recovered (0.09 mol, 69% yield). 9.537 g (1 equiv.) of sodium carbonate were added and the mixture was stirred overnight under reduced pressure. The mixture was subsequently filtrated. 7.11 g (0.063 mol, 48% yield) of the final product were recovered as colorless liquid by distillation under reduced pressure. The product crystallized at 6 °C.

**Figure 1:**
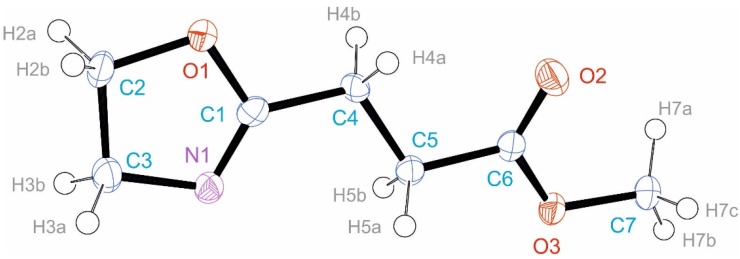
Asymmetric unit in the crystalline structure of **EstOx** (ORTEP drawing [33] with 50% probability ellipsoids). Selected bond lengths (Å): C1–N1: 1.263(4), C1–O1: 1.376(3), C2–O1: 1.458(3) C3–N1: 1.483(4), C2–C3: 1.529(4), C1–C4: 1.497(3), C4–C5: 1.515(4), C5–C6: 1.507(3), C6–O2: 1.211(3), C6–O3: 1.338(3), C7–O3: 1.448(3). Selected bond angles (°): N1–C1–O1: 118.6(2), N1–C1–C4: 128.9(2), O1–C1–C4: 112.5(2), O1–C2–C3: 104.0(2), N1–C3–C2: 105.1(2).

NMR (300 MHz, CDCl_3_): δ (ppm) = 2.55 (2 H, t, ^3^*J*_H–H_ = 6.6 Hz, H4a and H4b), 2.64 (2 H, t, ^3^*J*_H–H_ = 6.6 Hz, H5a and H5b), 3.67 (3 H, s, H7a, H7b and H7c), 3.78 (2 H, t, ^3^*J*_H–H_ = 9.3 Hz, H3a and H3b), 4.21 (2 H, t, ^3^*J*_H–H_ = 9.3 Hz, H2a and H2b) (for atomic labelling, see Figure 1).

IR (ATR, cm^−1^): ν = 2985 m, 2948 m, 2906 m, 2884 m, ν_str_ (CH); 1734 s, ν_str_ (C=O); 1669 s, ν_str_ (N=C); 1438 m, ν_str_ (CO); 1364 m, 1350 m, ν_def_ (CH_3_); 1204 m, 1161 s, ν_asym str_ (COCH_3_); 657 w, ν_bend_ (COCH_3_); 583 w, ν_def_ (CH_2_COCH_3_).

### 2.5. Preparation of 4-(2-Aminoethoxy)-4-oxobutanoic Acid **EstAA**

A solution of 0.5 g (0.0032 mol, 1 equiv.) of **EstOx** in 50 mL of methanol and 32 mL of an aqueous solution of sodium hydroxide (0.1 mol/L) were mixed and stirred for 1 h. The solvents were subsequently removed under reduced pressure. The crude product was dissolved in methanol, and 0.0032 mol of hydrochloric acid (aqueous 0.1 M solution) were added. After 10 min of stirring under reflux conditions, the mixture was stored overnight at 6 °C. The solvents were removed under reduced pressure, and 0.467 g (0.0029 mol, 91% yield) of the final product were recovered by recrystallization from ethanol.

^1^H NMR (20 °C, CDCl_3_, 300 MHz): δ (ppm) = 2.50 (2 H, t, ^3^*J*_H–H_ = 6.5 Hz, H3a and H3b), 2.66 (2 H, t, ^3^*J*_H–H_ = 6.5 Hz, H2a and H2b), 3.34 (2 H, t, ^3^*J*_H–H_ = 5.1 Hz, H6a and H6b), 4.38 (2 H, t, ^3^*J*_H–H_ = 5.1 Hz, H5a and H5b) (for atomic labelling, see Figure 3).

IR (ATR, cm^−1^): ν = 3438 m, ν_str_ (NH_2_); 2986 w, 2961 w, 2925 w, 2848 w, ν_str_ (CH); 2524 m ν_str_ (OH); 1729 s, ν_str_ (C=O); 1611 s, 1571 s, ν_def_ (NH_2_); 1312 s, ν_def_ (OH); 1248 s, 1155 s, ν_asym str_ (COC); 1013 s, ν_str_(NH_2_); 957 m, ν(COOH).

## 3. Results and Discussion

### 3.1. Synthesis of the Compounds **EstOx** and **EstAA**

**Scheme 3:**
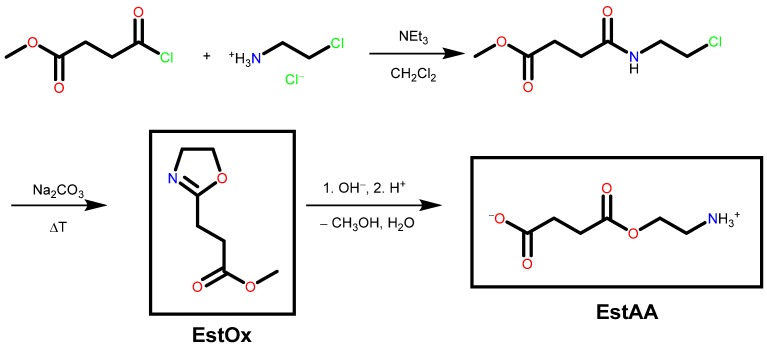
Synthesis of **EstOx** from methyl 4-chloro-4-oxobutanoate and chloroethylamine hydrochloride (**top**) as well as hydrolyses of the 2-oxazoline under alkaline and acidic conditions yielding **EstAA** (**bottom**).

Methyl 3-(4,5-dihydrooxazol-2-yl)propanoate **EstOx** can be prepared from the reaction of methyl 4-chloro-4-oxobutanoate and chloroethylamine hydrochloride and subsequent ring-closure under alkaline conditions (Scheme 3, top), following a literature protocol [32]. Hydrolyses of the ester bond and the 2-oxazoline ring yield the ester-functionalized amino acid 4-(2-aminoethoxy)-4-oxobutanoic acid **EstAA** (Scheme 3, bottom). Single crystals from both compounds were grown at 6 °C.

### 3.2. Crystal Structure of **EstOx**

**EstOx** crystallizes in the monoclinic space group *P*2_1_ with Z = 2 formula units in the unit cell. The asymmetric unit contains 1 formula unit (Figure 1). A detailed analysis of the dihedral angles reveals that the 2-oxazoline C_3_N_1_O_1_-pentacycle is almost planar [O1–C2–C3–N1: −4.2(3)°, N1–C1–O1–C2: −1.8(4)°, C3–C2–O1–C1: 3.6(3)°, O1–C1–N1–C3: −1.1(4)°, C2–C3–N1–C1: 3.3(3)°], and the carbon atoms of the side-chain are in *trans* alignment [C1–C4–C5–C6: −174.0(2)°]. Minor deviations of the overall *trans* alignment can only be observed around the ester group [C4–C5–C6–O2: 12.7(4)°, C4–C5–C6–O3: −168.4(2)°, C7–O3–C6–C5: −178.4(2)°].

Notably, like in the crystalline structures of 2-phenyl-2-oxazoline, 2-*^n^*nonyl-2-oxazoline and 2,2'-tetramethylenebis(2-oxazoline) [27], the two C–O bonds in the 2-oxazoline pentacycle differ significantly: While the C2–O1 bond with a length of 1.458(3) Å has the expected length of a C–O single bond [34,35], the C1–O1 bond with a length of 1.376(3) Å is significantly shorter and exhibits a value intermediate between the expected bond lengths of a C–O single and double bond. This phenomenon can be explained by the delocalization the π-electrons along the N–C–O segment of the C_3_N_1_O_1_-pentacycle. Along the O–C–O segment of the ester group of the side-chain, comparable delocalization can be observed: while the O2–C6 bond with a length of 1.211(3) exhibits a bond length typical for a C=O double bond, the O3–C6 bond with a length of 1.338(3) Å shows a value intermediate between the expected bond lengths of a C–O single and double bond.

Hence, the lengths of the C1–O1 bond and the O3–C6 bond are of very comparable value. While the potential (hetero) keto-enol tautomerism of the ester bond cannot be elucidated from the C–C bond lengths [C4–C5: 1.515(4) Å, C5–C6: 1.507(3) Å], it can be stated that the extent of π-electron delocalization along the N–C–O segment in 2-oxazolines is very comparable to that along the O–C–O segment in esters. The π-electron delocalization in esters is less pronounced than in amides (Scheme 2), but nonetheless significant: In 2-oxazolines, it renders the partial charge of the oxygen atom less negative, and the partial negative charge of the nitrogen atom more negative.

**Figure 2:**
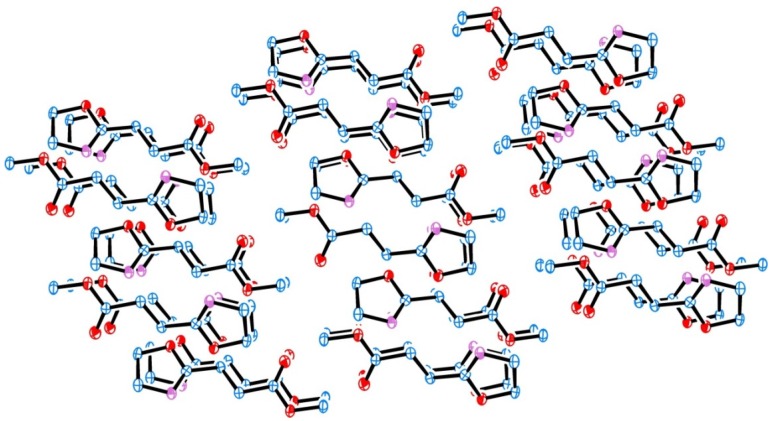
Arrangement of **EstOx** molecules in the crystalline phase.

Packing of the **EstOx** molecules in the crystalline phase seems to be controlled by steric factors only (Figure 2): The **EstOx** molecules are aligned in parallel fashion, with molecule-to-molecule distances of 5.547 Å; for comparison: Distances of adjacent molecules of 2,2'-tetramethylenebis(2-oxazoline) in the crystalline phase (that showed a packaging very similar to that of **EstOx**) exhibited a value of 5.084 Å [27].

### 3.3. Crystal Structure of **EstAA**

**Figure 3:**
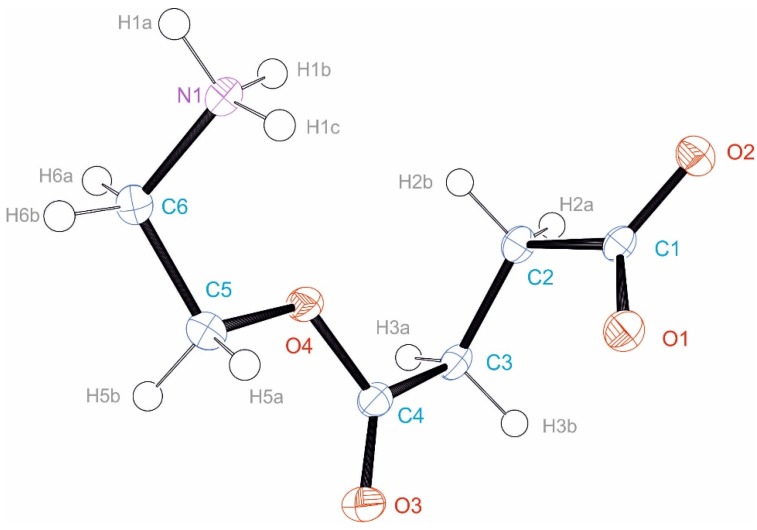
Asymmetric unit in the crystalline structure of **EstAA** (ORTEP drawing [33] with 50% probability ellipsoids). Selected bond lengths (Å): C1–O1: 1.251(3), C1–O2: 1.268(3), C4–O3: 1.205(4), C4–O4:1.354(3), C5–O4: 1.447(3), C6–N1: 1.489(3). Selected bond angles (°): O1–C1–C2 118.1(2), O3–C4–O4: 122.8(3), C1–C2–C3: 113.2(2).

**EstAA** crystallizes in its *zwitterionic* form in the monoclinic space group *P*2_1_/c with Z = 4 formula units in the unit cell. The asymmetric unit contains 1 formula unit (Figure 3). A detailed analysis of the dihedral angles shows that the C6–C5–O4–C4(O3)–C3 segment of the formula unit is in *trans* alignment [C4–O4–C5–C6: 179.6(2)°, C5–O4–C4–C3: 176.2(2)°, C5–O4–C4–O3: 0.2(4)°, while the ammonium group and the C2–C1(O1O2) segment deviate from that alignment [C1–C2–C3–C4: 68.7(3)°, O4–C5–C6–N1: −57.6(3)°]. In addition to its ester group with C–O bond lengths of 1.205(4) and 1.354(3) Å (which are almost identical to the C–O ester bond lengths of 1.211(3) and 1.338(3) Å in **EstOx**), **EstAA** also contains a carboxylate group that exhibits C–O bond lengths of 1.251(3) and 1.268(3) Å, which correspond in close proximity to the bond lengths of C–O double bonds [34,35]. The very minor difference among the two C–O bond lengths in the carboxylate group is assumed to originate from a different involvement of the oxygen atoms O1 and O2 atoms in the formation of hydrogen bonds (Table 2).

**Table 2:** Hydrogen bonds in the crystalline phase of **EstAA**.

Atom Names	Bond Length N–H	Distance H···O	Distance N···O	Angle NHO
N1–H1A···O2 ^A^	0.91 Å	1.84 Å	2.739(3) Å	171.1°
N1–H1B··O2 ^B^	0.91 Å	1.86 Å	2.741(3) Å	162.7°
N1–H1C··O1 ^C^	0.91 Å	1.84 Å	2.747(3) Å	172.2°

^A,B,C^: Symmetry operations used to generate equivalent atoms; A: −x + 2, y − 0.5, −z + 1.5; B: x, −y + 1.5, z − 0.5; C: −x + 2, −y + 1, −z + 2.

**Figure 4:**
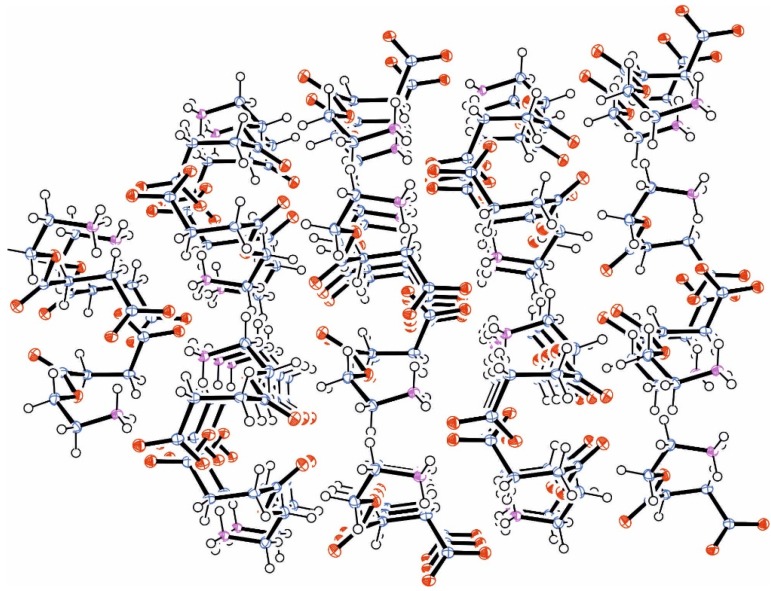
Arrangement of **EstAA** molecules in the crystalline phase. The orientation of the ammonium groups towards the carboxylate groups is indicative of hydrogen bond formation.

All acidic protons, namely the protons of the ammonium group, are involved in hydrogen bonds jointly with the oxygen atoms of the carboxylate group. The hydrogen bonds are likely to cause the deviation of the ammonium group and the carboxylate group from the overall *trans* alignment of the carbon chain of **EstAA**. The oxygen atoms of the ester group do not participate in the formation of hydrogen bonds. Correspondingly, packing of the **EstAA** molecules in the crystalline phase (Figure 4) is controlled by the formation of hydrogen bonds and large molecule-to-molecule distances of 8.001 Å.

### 3.4. Quantum Chemical Calculations of **EstOx**

In order to interpret the experimental findings, quantum chemical calculations were performed for the **EstOx** model system using the MRCC program [36,37]. Mulliken atomic charges [38] and Mayer bond orders [39] were computed with the density-fitting Hartree-Fock method using the correlation-consistent valence quadruple-zeta (cc-pVQZ) basis set [40] and the corresponding auxiliary basis sets [41]. The calculations were carried out at the experimental X-ray geometry of the molecule.

The theoretical results support the conjecture that the N–C–O segment has a delocalized electronic structure similar to an ester group. The calculated bond orders are 1.97 and 1.10, respectively, for the N=C and C–O bonds indicating that the former bond order is lower than a double bond, while the latter bond order is higher than a typical single bond. The computed partial charges for the nitrogen and oxygen atoms, namely −0.43 and −0.44, respectively, are comparable: despite the higher electronegativity of the oxygen atom (compared to the nitrogen atom), the charges of the hetero atoms are equalized due to the delocalization, and it also means that a cationic attack on the nitrogen atom is possible. It is interesting to compare the above numbers with the corresponding results for the ester group. The bond orders are 1.94 and 1.18 for the O=C and C–O bonds, while the partial charges of the carbonyl and other oxygen atom are −0.49 and −0.41, respectively, which demonstrate the similar electronic structure of the N–C–O and O–C–O segments.

## 4. Conclusions

The single crystal x-ray analysis of **EstOx** reveals that the π-electron delocalization along the N–C–O segment in 2-oxazolines occurs at a content comparable to the π-electron delocalization in esters along the O–C–O segment: while the observed C=N bond length in 2-oxazolines and the measured C=O bond length in the ester group are in good agreement with the literature values for the corresponding C=X double bonds [33,34], the C–O “single” bonds are similar to each other and exhibit a value intermediate between a single and double C–O bond. Quantum chemical calculations revealed calculated bond orders of 1.97 and 1.10 for the N=C and C–O bonds of the 2-oxazoline ring, and almost identical partial charges for the nitrogen and oxygen atoms of −0.43 and −0.44. As the charges of the hetero atoms are equalized due to the delocalization, the cationic attack on the nitrogen atom during the initiation of the cationic ring-opening polymerization is possible. The ester group exhibits bond orders of 1.94 and 1.18 for the O=C and C–O bonds, which demonstrates the similar electronic structure of the N–C–O and O–C–O segments.

## Supplementary Materials

Supplementary materials can be accessed at: http://www.mdpi.com/1996-1944/8/8/5385/s1.

## References

[1] Kappe C.O. (2004). Controlled microwave heating in modern organic synthesis. Angew. Chem. Int. Ed..

[2] Ebner C., Bodner T., Stelzer F., Wiesbrock F. (2011). One decade of microwave-assisted polymerizations: Quo vadis?. Macromol. Rapid Commun..

[3] Rossegger E., Schenk V., Wiesbrock F. (2013). Design strategies for functionalized poly(2-oxazoline)s and derived materials. Polymers.

[4] Kempe K., Becer C.R., Schubert U.S. (2011). Microwave-assisted polymerizations: Recent status and future perspectives. Macromolecules.

[5] Tomalia D.A., Sheetz D.P. (1966). Homopolymerization of 2-alkyl- and 2-aryl-2-oxazolines. J. Polym. Sci. A Polym. Chem..

[6] Seeliger W., Aufderhaar E., Diepers W., Feinauer R., Nehring R., Thier W., Hellmann H. (1966). Recent syntheses and reactions of cyclic imidic esters. Angew. Chem. Int. Ed..

[7] Kagiya T., Narisawa S., Maeda T., Fukui K. (1966). Ring-opening polymerisation of 2-substituted 2-oxazolines. J. Polym. Sci. B Polym. Lett..

[8] Bassiri T.G., Levy A., Litt M. (1967). Polymerization of cyclic imino ethers. I. Oxazolines. J. Polym. Sci. B Polym. Lett..

[9] Hoogenboom R. (2009). Poly(2-oxazoline)s: A polymer class with numerous potential applications. Angew. Chem. Int. Ed..

[10] Hoogenboom R., Schlaad H. (2011). Bioinspired poly(2-oxazoline)s. Polymers.

[11] Schlaad H., Diehl C., Gress A., Meyer M., Demirel A.L., Nur Y., Bertin A. (2010). Poly(2-oxazoline)s as smart bioinspired polymers. Macromol. Rapid Commun..

[12] Gaertner F.C., Luxenhofer R., Blechert B., Jordan R., Essler M. (2007). Synthesis, biodistribution and excretion of radiolabeled poly(2-alkyl-2-oxazoline)s. J. Control. Release.

[13] Waschinski C.J., Tiller J.C. (2005). Poly(oxazoline)s with telechelic antimicrobial functions. Biomacromolecules.

[14] Waschinski C.J., Barnert S., Theobald A., Schubert R., Kleinschmidt F., Hoffmann A., Saalwächter K., Tiller J.C. (2008). Insights in the antibacterial action of poly(methyloxazoline)s with a biocidal end group and varying satellite groups. Biomacromolecules.

[15] Kelly A., Kaltenhauser V., Mühlbacher I., Rametsteiner K., Kren H., Slugovc C., Stelzer F., Wiesbrock F. (2013). Poly(2-oxazoline)-derived contact biocides: Contributions to the understanding of antimicrobial activity. Macromol. Biosci..

[16] Kelly A.M., Wiesbrock F. (2012). Strategies for the synthesis of poly(2-oxazoline)-based hydrogels. Macromol. Rapid Commun..

[17] Schenk V., Ellmaier L., Rossegger E., Edler M., Griesser T., Weidinger G., Wiesbrock F. (2012). Water-developable poly(2-oxazoline)-based negative photoresists. Macromol. Rapid Commun..

[18] Fijten M.W.M., Haensch C., van Lankvelt B.M., Hoogenboom R., Schubert U.S. (2008). Clickable poly(2-oxazoline)s as versatile building blocks. Macromol. Chem. Phys..

[19] Zschoche S., Rueda J., Boyko V., Krahl F., Arndt K.-F., Voit B. (2011). Thermo-responsive nanogels based on poly[NIPAAm-graft-(2-alkyl-2-oxazoline)]s crosslinked in the micellar state. Macromol. Chem. Phys..

[20] Kempe K., Hoogenboom R., Jaeger M., Schubert U.S. (2011). Three-fold metal-free efficient “click” reactions onto a multifunctional poly(2-oxazoline) designer scaffold. Macromolecules.

[21] Kelly A.M., Hecke A., Wirnsberger B., Wiesbrock F. (2011). Synthesis of poly(2-oxazoline)-based hydrogels with tailor-made swelling degrees capable of stimuli-triggered compound release. Macromol. Rapid Commun..

[22] Ten Brummelhuis N., Schlaad H. (2011). Stimuli-responsive star polymers through thiol-yne core functionalization/crosslinking of block copolymer micelles. Polym. Chem..

[23] Wiesbrock F., Hoogenboom R., Leenen M.A.M., Meier M.A.R., Schubert U.S. (2005). Investigation of the living cationic ring-opening polymerization of 2-methyl-, 2-ethyl-, 2-nonyl-, and 2-phenyl-2-oxazoline in a single-mode microwave reactor. Macromolecules.

[24] Hoogenboom R., Fijten M.W.M., Kickelbick G., Schubert U.S. (2010). Synthesis and crystal structures of multifunctional tosylates as basis for star-shaped poly(2-ethyl-2-oxazoline)s. Beilstein J. Org. Chem..

[25] Luxenhofer R., Bezen M., Jordan R. (2008). Kinetic investigations on the polymerization of 2-oxazolines using pluritriflate initiators. Macromol. Rapid Commun..

[26] Wiesbrock F., Hoogenboom R., Abeln C.H., Schubert U.S. (2004). Single-mode microwave ovens as new reaction devices: Accelerating the living polymerization of 2-ethyl-2-oxazoline. Macromol. Rapid Commun..

[27] Bodner T., Ellmaier L., Schenk V., Albering J., Wiesbrock F. (2011). Delocalized π-electrons in 2-oxazoline rings resulting in negatively charged nitrogen atoms: Revealing the selectivity during the initiation of cationic ring-opening polymerizations. Polym. Int..

[28] (1997). SHELXS-97.

[29] (1997). SHELXL-97.

[30] Spek A.L. (2003). Single-crystal structure validation with the program PLATON. J. Appl. Crystallogr..

[31] (1996). Area-Detector Absorption Correction.

[32] Zarka M.T., Nuyken O., Weberskirch R. (2003). Amphiphilic polymer supports for the asymmetric hydrogenation of amino acid precursors in water. Chem. Eur. J..

[33] Johnson C.K. (1976). ORTEP.

[34] Cottrell T.L. (1958). The Strengths of Chemical Bonds.

[35] Benson S.W. (1965). III - Bond energies. J. Chem. Educ..

[36] Kállay M., Rolik Z., Csontos J., Ladjánszki I., Szegedy L., Ladóczki B., Samu G., MRCC A Quantum Chemical Program Suite. http://www.mrcc.hu.

[37] Rolik Z., Szegedy L., Ladjánszki I., Ladóczki B., Kállay M. (2013). An efficient linear-scaling CCSD(T) method based on local natural orbitals. J. Chem. Phys..

[38] Mulliken R.S. (1955). Electronic population analysis on LCAO-MO molecular wave functions. J. Chem. Phys..

[39] Mayer I. (1983). Charge, bond order and valence in the ab initio SCF theory. Chem. Phys. Lett..

[40] Dunning T.H. (1989). Gaussian basis sets for use in correlated molecular calculations. I. The atoms boron through neon and hydrogen. J. Chem. Phys..

[41] Weigend F. (2008). Hartree-Fock exchange fitting basis sets for H to Rn. J. Comp. Chem..

