# Supplementary material for: The π-Electron Delocalization in 2-Oxazolines Revisited: Quantification and Comparison with Its Analogue in Esters
Source: Materials (Basel). 2015 Aug 21;8(8):5385–97. doi: 10.3390/ma8085249 (PMC5295637; doi:10.3390/ma8085249)
Supplement: Supplementary file 1 [file materials-08-05249-s001.pdf]

Supplementary Information

X-ray Analysis of Methyl-3-(4,5-dihydrooxazol-2-yl)propanoate, EstOx

Table S1. Crystal data and structure refinement for methyl-3-(4,5-dihydrooxazol-2-yl)propanoate, **EstOx**.

Note	Value
Identification code	methyl-3-(4,5-dihydrooxazol-2-yl)propanoate, EstOx
Empirical formula	C7 H11 N O3
Formula weight	157.07
Temperature	100(2) K
Wavelength	0.71073 Å
Crystal system	Monoclinic
Space group	$P2_1$
Unit cell dimensions	a = 5.547(2) Å, α = 90°
	b = 6.765(3) Å, β = 91.583(13)°
	c = 9.993(4) Å, γ = 90°
Volume	374.9(3) $Å^3$
Z	2
Density (calculated)	1.391 Mg/m^3
Absorption coefficient	0.109 mm^{-1}
F(000)	168
Crystal size	0.31 × 0.24 × 0.19 mm^3
Theta range for data collection	2.04 to 26.00°
Index ranges	−6<=h<=6, −7<=k<=8, −12<=l<=12
Reflections collected	4559
Independent reflections	1278 [R_{int} = 0.0659]
Completeness to theta = 26.00°	98.5%
Absorption correction	None
Max. and min. transmission	0.9808 and 0.9690
Refinement method	Full-matrix least-squares on F^2
Data / restraints / parameters	1278/1/101
Goodness-of-fit on F2	1.069
Final R indices [I>2sigma(I)]	$R1$ = 0.0530, $wR2$ = 0.1352
R indices (all data)	$R1$ = 0.0601, $wR2$ = 0.1407
Absolute structure parameter	1(2)
Largest diff. peak and hole	0.306 and −0.219 e.$Å^{-3}$

Table S2. Atomic coordinates (×10^4) and equivalent isotropic displacement parameters (Å^2 × 10^3) for methyl-3-(4,5-dihydrooxazol-2-yl)propanoate, **EstOx**. U(eq) is defined as one third of the trace of the orthogonalized U^{ij} tensor.

Note	x	y	z	U(eq)
C(1)	6684(5)	7964(4)	10649(3)	19(1)
C(2)	4258(5)	7658(5)	12415(3)	22(1)
C(3)	6542(5)	8832(5)	12773(3)	25(1)
O(3)	12091(3)	8842(3)	6952(2)	22(1)
O(1)	4473(3)	7229(3)	10994(2)	22(1)
O(2)	8356(4)	7735(4)	6497(2)	29(1)
N(1)	7956(4)	8836(4)	11534(2)	21(1)
C(4)	7235(5)	7578(5)	9215(3)	18(1)
C(5)	9539(5)	8564(5)	8782(3)	20(1)
C(7)	12561(5)	8701(5)	5537(2)	25(1)
C(6)	9871(5)	8311(4)	7301(2)	18(1)

Table S3. Bond lengths [Å] and angles [°] for methyl-3-(4,5-dihydrooxazol-2-yl)propanoate, **EstOx**.

Note	Value
C(1)-N(1)	1.263(4)
C(1)-O(1)	1.376(3)
C(1)-C(4)	1.497(3)
C(2)-O(1)	1.458(3)
C(2)-C(3)	1.529(4)
C(2)-H(2A)	0.9900
C(2)-H(2B)	0.9900
C(3)-N(1)	1.483(4)
C(3)-H(3A)	0.9900
C(3)-H(3B)	0.9900
O(3)-C(6)	1.338(3)
O(3)-C(7)	1.448(3)
O(2)-C(6)	1.211(3)
C(3)-H(3A)	0.9900
C(3)-H(3B)	0.9900
O(3)-C(6)	1.338(3)
O(3)-C(7)	1.448(3)
O(2)-C(6)	1.211(3)
C(3)-H(3A)	0.9900
C(3)-H(3B)	0.9900
O(3)-C(6)	1.338(3)
O(3)-C(7)	1.448(3)
O(2)-C(6)	1.211(3)
C(4)-C(5)	1.515(4)
C(4)-H(4A)	0.9900
C(4)-H(4B)	0.9900
C(5)-C(6)	1.507(3)
C(5)-H(5A)	0.9900
C(5)-H(5B)	0.9900

Table S3. *Cont.*

Note	Value
C(7)-H(7A)	0.9800
C(7)-H(7B)	0.9800
C(7)-H(7C)	0.9800
N(1)-C(1)-O(1)	118.6(2)
N(1)-C(1)-C(4)	128.9(2)
O(1)-C(1)-C(4)	112.5(2)
O(1)-C(2)-C(3)	104.0(2)
O(1)-C(2)-H(2A)	111.0
C(3)-C(2)-H(2A)	111.0
O(1)-C(2)-H(2B)	111.0
C(3)-C(2)-H(2B)	111.0
H(2A)-C(2)-H(2B)	109.0
N(1)-C(3)-C(2)	105.1(2)
N(1)-C(3)-H(3A)	110.7
C(2)-C(3)-H(3A)	110.7
N(1)-C(3)-H(3B)	110.7
C(2)-C(3)-H(3B)	110.7
H(3A)-C(3)-H(3B)	108.8
C(6)-O(3)-C(7)	115.38(19)
C(1)-O(1)-C(2)	105.6(2)
C(1)-N(1)-C(3)	106.6(2)
C(1)-C(4)-C(5)	113.1(2)
C(1)-C(4)-H(4A)	109.0
C(5)-C(4)-H(4A)	109.0
C(1)-C(4)-H(4B)	109.0
C(5)-C(4)-H(4B)	109.0
H(4A)-C(4)-H(4B)	107.8
C(6)-C(5)-C(4)	110.9(2)
C(6)-C(5)-H(5A)	109.5
C(4)-C(5)-H(5A)	109.5
C(6)-C(5)-H(5B)	109.5
C(4)-C(5)-H(5B)	109.5
H(5A)-C(5)-H(5B)	108.0
O(3)-C(7)-H(7A)	109.5
O(3)-C(7)-H(7B)	109.5
H(7A)-C(7)-H(7B)	109.5
O(3)-C(7)-H(7C)	109.5
H(7A)-C(7)-H(7C)	109.5
H(7B)-C(7)-H(7C)	109.5
O(2)-C(6)-O(3)	122.7(2)
O(2)-C(6)-C(5)	125.9(2)
O(3)-C(6)-C(5)	111.3(2)

Table S4. Anisotropic displacement parameters (Å^2 × 10^3) for ethyl-3-(4,5-dihydrooxazol-2-yl)propanoate, **EstOx**. The anisotropic displacement factor exponent takes the form: $-2\ ^2[h^2\ a^{*2}U^{11} + ... + 2\ h\ k\ a^*\ b^*\ U^{12}]$.

Note	U^{11}	U^{22}	U^{33}	U^{23}	U^{13}	U^{12}
C(1)	21(1)	12(2)	23(1)	1(1)	3(1)	2(1)
C(2)	29(2)	20(2)	18(1)	2(1)	8(1)	0(1)
C(3)	34(2)	20(2)	20(1)	−1(1)	6(1)	0(2)
O(3)	27(1)	23(1)	18(1)	0(1)	5(1)	−2(1)
O(1)	24(1)	23(1)	19(1)	−2(1)	4(1)	−4(1)
O(2)	30(1)	37(2)	21(1)	−5(1)	2(1)	−3(1)
N(1)	27(1)	18(1)	19(1)	0(1)	3(1)	−1(1)
C(4)	25(1)	13(2)	17(1)	−4(1)	2(1)	1(1)
C(5)	25(1)	16(2)	18(1)	0(1)	2(1)	0(1)
C(7)	30(2)	29(2)	17(1)	0(1)	6(1)	−3(2)
C(6)	24(1)	13(2)	18(1)	0(1)	3(1)	4(1)

Table S5. Hydrogen coordinates (×10^4) and isotropic displacement parameters (Å^2 × 10^3) for methyl-3-(4,5-dihydrooxazol-2-yl)propanoate, **EstOx**.

Note	x	y	z	U(eq)
H(2A)	4189	6422	12944	27
H(2B)	2795	8450	12579	27
H(3A)	6134	10198	13041	29
H(3B)	7461	8191	13517	29
H(4A)	5875	8061	8641	22
H(4B)	7373	6134	9076	22
H(5A)	9480	9990	9001	24
H(5B)	10931	7977	9280	24
H(7A)	11930	7445	5187	38
H(7B)	14304	8763	5405	38
H(7C)	11769	9800	5061	38

Table S6. Torsion angles [°] for methyl-3-(4,5-dihydrooxazol-2-yl)propanoate, **EstOx**.

Note	Value
O(1)-C(2)-C(3)-N(1)	−4.2(3)
N(1)-C(1)-O(1)-C(2)	−1.8(4)
C(4)-C(1)-O(1)-C(2)	177.9(2)
C(3)-C(2)-O(1)-C(1)	3.6(3)
O(1)-C(1)-N(1)-C(3)	−1.1(4)
C(4)-C(1)-N(1)-C(3)	179.3(3)
C(2)-C(3)-N(1)-C(1)	3.3(3)
N(1)-C(1)-C(4)-C(5)	−6.4(4)
O(1)-C(1)-C(4)-C(5)	173.9(2)
C(1)-C(4)-C(5)-C(6)	−174.0(2)
C(7)-O(3)-C(6)-O(2)	0.5(4)
C(7)-O(3)-C(6)-C(5)	−178.4(2)
C(4)-C(5)-C(6)-O(2)	12.7(4)
C(4)-C(5)-C(6)-O(3)	−168.4(2)

X-ray Analysis of 4-(2-aminoethoxy)-4-oxobutanoic acid, EstAA

Table S7. Crystal data and structure refinement for 4-(2-aminoethoxy)-4-oxobutanoic acid, **EstAA**.

Note	Value
Identification code	4-(2-aminoethoxy)-4-oxobutanoic acid, EstAA
Empirical formula	C6 H11 N O4
Formula weight	161.16
Temperature	100(2) K
Wavelength	0.71073 Å
Crystal system	Monoclinic
Space group	$P2_1/c$
Unit cell dimensions	a = 10.3132(8) Å, α = 90°
	b = 9.0304(7) Å, β = 96.688(5)°
	c = 8.0012(7) Å, γ = 90°
Volume	740.10(10) $Å^3$
Z	4
Density (calculated)	1.446 Mg/m^3
Absorption coefficient	0.122 mm^{-1}
F(000)	344
Crystal size	0.36 × 0.26 × 0.12 mm^3
Theta range for data collection	1.99 to 28.03°
Index ranges	0<=h<=13, −11<=k<=0, −10<=l<=10
Reflections collected	1786
Independent reflections	1786 [R_{int} = 0.0000]
Completeness to theta = 26.00°	100.0%
Absorption correction	Semi-empirical from equivalents
Max. and min. transmission	0.9855 and 0.9574
Refinement method	Full-matrix least-squares on F2
Data/restraints/parameters	1786/0/101
Goodness-of-fit on F2	1.255
Final R indices [I>2sigma(I)]	$R1$ = 0.0630, $wR2$ = 0.1432
R indices (all data)	$R1$ = 0.0736, $wR2$ = 0.1467
Largest diff. peak and hole	0.362 and −0.325 e.$Å^{-3}$

Table S8. Atomic coordinates (×10[4]) and equivalent isotropic displacement parameters ($Å^2 \times 10^3$) for 4-(2-aminoethoxy)-4-oxobutanoic acid, EstAA. U(eq) is defined as one third of the trace of the orthogonalized Uij tensor.

Note	x	y	z	U(eq)
O(2)	10396(2)	7183(2)	11254(2)	18(1)
C(2)	8534(3)	5625(3)	11313(3)	16(1)
O(1)	8479(2)	8008(2)	10024(3)	20(1)
C(1)	9178(3)	7055(3)	10826(3)	15(1)
O(3)	5464(2)	6624(2)	8917(3)	22(1)
C(3)	7043(3)	5726(3)	11161(3)	16(1)
O(4)	6897(2)	4867(2)	8361(2)	18(1)
C(4)	6382(3)	5843(3)	9385(4)	16(1)
C(5)	6280(3)	4813(3)	6643(4)	19(1)
C(6)	7009(3)	3673(3)	5738(4)	18(1)
N(1)	8423(2)	4036(3)	5820(3)	15(1)

Table S9. Bond lengths [Å] and angles [°] for 4-(2-aminoethoxy)-4-oxobutanoic acid, **EstAA**.

Note	Value
O(2)-C(1)	1.268(3)
C(2)-C(1)	1.524(4)
C(2)-C(3)	1.531(4)
C(2)-H(2A)	0.9900
C(2)-H(2B)	0.9900
O(1)-C(1)	1.251(3)
O(3)-C(4)	1.205(4)
C(3)-C(4)	1.506(4)
C(3)-H(3A)	0.9900
C(3)-H(3B)	0.9900
O(4)-C(4)	1.354(3)
O(4)-C(5)	1.447(3)
C(5)-C(6)	1.509(4)
C(5)-H(5A)	0.9900
C(5)-H(5B)	0.9900
C(6)-N(1)	1.489(3)
C(6)-H(6A)	0.9900
C(6)-H(6B)	0.9900
N(1)-H(1A)	0.9100
N(1)-H(1B)	0.9100
N(1)-H(1C)	0.9100
C(1)-C(2)-C(3)	113.2(2)
C(1)-C(2)-H(2A)	108.9
C(3)-C(2)-H(2A)	108.9
C(1)-C(2)-H(2B)	108.9
C(3)-C(2)-H(2B)	108.9
H(2A)-C(2)-H(2B)	107.8

Table S9. *Cont.*

Note	Value
O(1)-C(1)-O(2)	124.7(3)
O(1)-C(1)-C(2)	118.1(2)
O(2)-C(1)-C(2)	117.1(2)
C(4)-C(3)-C(2)	114.8(2)
C(4)-C(3)-H(3A)	108.6
C(2)-C(3)-H(3A)	108.6
C(4)-C(3)-H(3B)	108.6
C(2)-C(3)-H(3B)	108.6
H(3A)-C(3)-H(3B)	107.6
C(4)-O(4)-C(5)	115.9(2)
O(3)-C(4)-O(4)	122.8(3)
O(3)-C(4)-C(3)	126.0(3)
O(4)-C(4)-C(3)	111.0(2)
O(4)-C(5)-C(6)	106.9(2)
O(4)-C(5)-H(5A)	110.3
C(6)-C(5)-H(5A)	110.3
O(4)-C(5)-H(5B)	110.3
C(6)-C(5)-H(5B)	110.3
H(5A)-C(5)-H(5B)	108.6
N(1)-C(6)-C(5)	111.6(2)
N(1)-C(6)-H(6A)	109.3
C(5)-C(6)-H(6A)	109.3
N(1)-C(6)-H(6B)	109.3
C(5)-C(6)-H(6B)	109.3
H(6A)-C(6)-H(6B)	108.0
C(6)-N(1)-H(1A)	109.5
C(6)-N(1)-H(1B)	109.5
H(1A)-N(1)-H(1B)	109.5
C(6)-N(1)-H(1C)	109.5
H(1A)-N(1)-H(1C)	109.5
H(1B)-N(1)-H(1C)	109.5

Table S10. Anisotropic displacement parameters ($Å^2 \times 10^3$) for 4-(2-aminoethoxy)-4-oxobutanoic acid, EstAA. The anisotropic displacement factor exponent takes the form: $-2\ ^{2}[\ h^2\ a^{*2}U^{11} + ... + 2\ h\ k\ a^*\ b^*\ U^{12}]$.

Note	U^{11}	U^{22}	U^{33}	U^{23}	U^{13}	U^{12}
O(2)	18(1)	19(1)	19(1)	3(1)	2(1)	−2(1)
C(2)	20(1)	13(1)	15(1)	1(1)	3(1)	0(1)
O(1)	21(1)	15(1)	23(1)	4(1)	1(1)	0(1)
C(1)	18(1)	15(1)	12(1)	−3(1)	3(1)	1(1)
O(3)	18(1)	20(1)	29(1)	−2(1)	4(1)	4(1)
C(3)	21(1)	13(1)	17(1)	−3(1)	6(1)	−1(1)
O(4)	17(1)	20(1)	16(1)	−3(1)	1(1)	3(1)
C(4)	15(1)	14(1)	21(1)	−2(1)	8(1)	−2(1)
C(5)	16(1)	20(1)	20(1)	0(1)	0(1)	1(1)
C(6)	16(1)	18(1)	18(1)	−2(1)	1(1)	−1(1)
N(1)	18(1)	13(1)	14(1)	−1(1)	3(1)	1(1)

Table S11. Hydrogen coordinates (×10^4) and isotropic displacement parameters (Å^2 × 10^3) for 4-(2-aminoethoxy)-4-oxobutanoic acid, EstAA.

Note	x	y	z	U(eq)
H(2A)	8868	5372	12488	19
H(2B)	8783	4814	10581	19
H(3A)	6705	4837	11692	20
H(3B)	6800	6601	11802	20
H(5A)	6328	5794	6101	22
H(5B)	5350	4530	6614	22
H(6A)	6906	2689	6252	21
H(6B)	6627	3621	4545	21
H(1A)	8790	3488	5043	23
H(1B)	8825	3824	6866	23
H(1C)	8519	5016	5600	23

Table S12. Torsion angles [°] for 4-(2-aminoethoxy)-4-oxobutanoic acid, EstAA.

Note	Value
C(3)-C(2)-C(1)-O(1)	−15.6(3)
C(3)-C(2)-C(1)-O(2)	165.1(2)
C(1)-C(2)-C(3)-C(4)	68.7(3)
C(5)-O(4)-C(4)-O(3)	0.2(4)
C(5)-O(4)-C(4)-C(3)	176.2(2)
C(2)-C(3)-C(4)-O(3)	−139.5(3)
C(2)-C(3)-C(4)-O(4)	44.7(3)
C(4)-O(4)-C(5)-C(6)	179.6(2)
O(4)-C(5)-C(6)-N(1)	−57.6(3)

Table S13. Hydrogen bonds for 4-(2-aminoethoxy)-4-oxobutanoic acid, EstAA [Å and °].

D-H...A	d(D-H)	d(H...A)	d(D...A)	<(DHA)
N(1)-H(1B)...O(2)#1	0.91	1.86	2.741(3)	162.7
N(1)-H(1C)...O(1)#2	0.91	1.84	2.747(3)	172.2
N(1)-H(1A)...O(2)#3	0.91	1.84	2.739(3)	171.1

Symmetry transformations used to generate equivalent atoms: #1 −x+2, −y+1, −z+2, #2 x, −y+3/2, z−1/2, #3 −x+2, y−1/2, −z+3/2.
